# Good fences make for good neighbors but bad science: a review of what improves Bayesian reasoning and why

**DOI:** 10.3389/fpsyg.2015.00340

**Published:** 2015-03-31

**Authors:** Gary L. Brase, W. Trey Hill

**Affiliations:** ^1^Department of Psychological Sciences, Kansas State UniversityManhattan, KS, USA; ^2^Department of Psychology, Fort Hays State UniversityHays, KS, USA

**Keywords:** Bayesian reasoning, frequencies, probabilities, ecological rationality, heuristics and biases, pictorial aids, numeracy

## Abstract

Bayesian reasoning, defined here as the updating of a posterior probability following new information, has historically been problematic for humans. Classic psychology experiments have tested human Bayesian reasoning through the use of word problems and have evaluated each participant’s performance against the normatively correct answer provided by Bayes’ theorem. The standard finding is of generally poor performance. Over the past two decades, though, progress has been made on how to improve Bayesian reasoning. Most notably, research has demonstrated that the use of frequencies in a natural sampling framework—as opposed to single-event probabilities—can improve participants’ Bayesian estimates. Furthermore, pictorial aids and certain individual difference factors also can play significant roles in Bayesian reasoning success. The mechanics of how to build tasks which show these improvements is not under much debate. The explanations for *why* naturally sampled frequencies and pictures help Bayesian reasoning remain hotly contested, however, with many researchers falling into ingrained “camps” organized around two dominant theoretical perspectives. The present paper evaluates the merits of these theoretical perspectives, including the weight of empirical evidence, theoretical coherence, and predictive power. By these criteria, the ecological rationality approach is clearly better than the heuristics and biases view. Progress in the study of Bayesian reasoning will depend on continued research that honestly, vigorously, and consistently engages across these different theoretical accounts rather than staying “siloed” within one particular perspective. The process of science requires an understanding of competing points of view, with the ultimate goal being integration.

## Introduction

Imagine, for one moment, the following scene: A !Kung woman begins her day by foraging for berries in the Kalahari Desert. Wandering from patch to patch, she searches for substantial portions of subsistence. Foraging is not always fruitful; it does not always yield food, and sometimes it does not yield enough food to justify the calories expended during the act of foraging. Foragers must decipher patterns from the environment in order to be successful and efficient. For example, the !Kung woman may have success 90% of the time she travels to the east canyon, but only when she forages in springtime. During the summer months, the east canyon may be barren of food. At some level of cognition, the woman must coarsely analyze the data from her travels in order to determine the odds of finding food in the east canyon, given the fact that it is springtime or summer. From a psychological perspective, we may wonder what is happening at the cognitive, or algorithmic, level in the woman’s mind. How is she storing the information, and how is she arriving at seemingly appropriate solutions to this particular problem of calculating a posterior probability of finding food given certain environmental cues? Although the surface of this paper provides guidance for ways to improve Bayesian reasoning, it also delves into the deeper questions of how and why the mind is designed to solve certain problems with specific inputs.

## The General Case of Bayesian Reasoning

The technical name for what the !Kung woman is doing in the above story is *Bayesian reasoning.* Although Bayesian reasoning sometimes has a narrow mathematical definition (i.e., the use of Bayes theorem, specifically), for the purposes of psychological research the more relevant definition is the general process of using new information (e.g., season of the year) to calculate the revised likelihood that an event of a known prior base rate will occur (e.g., successfully finding food). Humans have, historically, needed to perform quick computational estimates of such probabilities in order to navigate various aspects of ancestral environments (Cosmides and Tooby, 1996). Therefore, it seems scientifically unproductive to insist on the narrow definition (in that an explicit Bayes theorem is only a few centuries old) in describing human judgments and decision making. It is important therefore to distinguish between a narrow and rigid usage of “applying Bayes’ theorem” in defining Bayesian reasoning, as compared to a more general usage of Bayesian reasoning as a process of adaptively updating prior probabilities with new information (by whatever means) to reach a new, or posterior, probability. This more general definition of Bayesian reasoning, which is the sensible one to take from the perspective of a cognitive psychologist, is to evaluate behaviors as the potential product of cognitive mechanisms acting “as if” they were Bayesian. Specifically, this general definition of Bayesian reasoning can be used to classify behaviors based on the observable evidence that the individual organism in question used new evidence to update its estimate that an event would occur. Often, this is ultimately tested through some measurable behavior (e.g., a decision to act in accordance with this new evidence’s implications for the posterior probability of an event).

## Bayesian Reasoning as a Serious, Real World Problem

Traditional research on people’s abilities to engage in Bayesian reasoning uses the following protocol: a person is presented with a description of a situation in which Bayesian reasoning is relevant, the necessary numerical information for Bayesian calculations, and then a request that the participant calculate the posterior probability (expressed in terms of the relevant situation). For example, one such task (adapted from Chapman and Liu, 2009) is as follows:

The serum test screens pregnant women for babies with Down’s syndrome. The test is a very good one, but not perfect. Roughly 5% of babies have Down’s syndrome. If a baby has Down’s syndrome, there is a 80% chance that the result will be positive. If the baby is unaffected, there is still a 20% chance that the result will still be positive. A pregnant woman has been tested and the result is positive. What is the chance that her baby actually has Down’s syndrome?

Undergraduates, medical students, and even physicians do quite poorly on this type of Bayesian reasoning task (e.g., Casscells et al., 1978; Gigerenzer et al., 2007), including when it is in a medical testing context such as the above example. Such failures of Bayesian reasoning suggest potentially tragic consequences for medical decision making, as well as any other real world topics that involve similar calculations.

Interestingly, evaluations of how and why people do poorly in Bayesian reasoning has changed over the years. In the early days of research on Bayesian reasoning, the dominant view by researchers was that humans were approximating Bayes’ theorem, but erred in being far too conservative in their estimates (e.g., Edwards, 1982). That is, people did not utilize the new information as much as they should; relying too much on the base rate information. Later work, however, shifted to the idea that the dominant error was in the opposite direction: that people generally erred in relying too much on the new information and neglecting the base rate, either partially or entirely (e.g., Kahneman and Tversky, 1972; Tversky and Kahneman, 1974, 1982). This later approach is one of the better known positions within what is known as the *heuristics and biases* paradigm, within which base rate neglect was considered so strong and pervasive that at one point it was asserted: “In his evaluation of evidence, man is apparently not a conservative Bayesian: he is not Bayesian at all” (Kahneman and Tversky, 1972, p. 450).

## Improving Bayesian Reasoning

Nevertheless, research continued on human Bayesian reasoning and how to improve it. Beginning in the 1990s, progress began to occur, followed quickly by theoretical debates. There continue to be disagreements to this day, but there now clearly are certain procedures which do in fact improve human Bayesian reasoning. These include: using a natural sampling structure, using frequencies, and using pictures. Each of these procedures also raise theoretical issues about what cognitive processes underlying the improvement in human reasoning, and this paper will look at each of these in turn. We will also look at the role of individual differences in aptitude and motivation within the context of Bayesian reasoning before concluding with an overall assessment.

### Natural Sampling and Frequencies in Bayesian Reasoning

A seminal paper in terms of improving Bayesian reasoning and the current issues revolving around those improvements is Gigerenzer and Hoffrage (1995). This paper described a structure for presenting information in such a way that it greatly helped people reach correct Bayesian conclusions. This structure is one of whole-number frequencies in a natural sampling framework. (This original paper used the unfortunately ambiguous label of “frequency format” for this structure, which has led to some confusion; see Gigerenzer and Hoffrage, 1999, 2007; Lewis and Keren, 1999; Mellers and McGraw, 1999; Vranas, 2000; Gigerenzer, 2001.) There are thus two aspect of this structure: (a) the use of frequencies as a numerical format, and (b) the use of a particular structure, called natural sampling, for the relationships between the numbers. The rationale for both of these aspects is similar: they map onto the type of information which the human mind generally encounters in the natural environment, both currently and over evolutionary history. For this reason, the Gigerenzer and Hoffrage position is often described as the* ecological rationality* approach.

**FIGURE 1:**
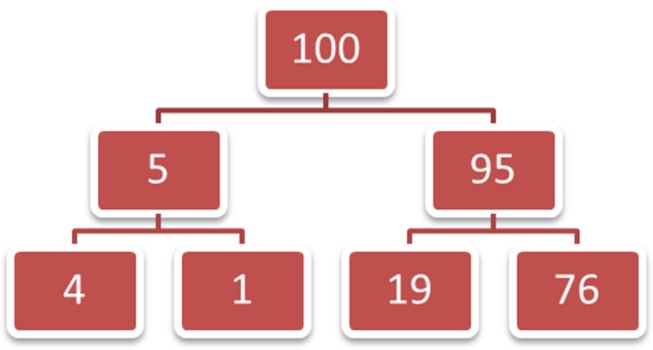
**An illustration of a natural sampling framework: the total population (100) is categorized into groups (5/95) and those groups are categorized into parallel sub-groups below that**.

It can be challenging to dissociate natural sampling from frequencies. When considering the occurrence of objects or events in the real world, that experience tends to strongly imply frequency counts as the format in which that information would be encoded. The actual format of natural sampling, however, is actually the online categorization of that information into groups, including groups which can be subsets of one another. **Figure 1** shows the previously given Bayesian reasoning task information (about a Down’s syndrome serum test) as naturally sampled frequencies. In this case we imagine (or recall) 100 experiences with this test, and five of those experiences were with a baby who had Down’s syndrome (i.e., 5% base rate). Those five experiences can be further categorized by when the test came out positive (4 times; 4 out of 5 is 80%), and the 95 cases of babies without Down’s syndrome can be similarly categorized by the test results (19 false positive results; 19 out of 95 is 20%). This nested categorization structure creates numbers in the lower-most row for which the base-rates (from the initial categorization groups) are automatically taken into account already. This, in turn, makes the calculations for Bayesian reasoning less computationally difficult. (Specifically, the probabilistic version of Bayes theorem is p(H|D) =p(H)p(D|H)/p(H)p(D|H) + p(∼H)p(D|∼H), with D = new data and H = the hypothesis, whereas with natural sampling this equation can be simplified to p(H|D) = d&h/d&h + d&∼h, with d&h = frequency of data and the hypothesis and d&∼h = frequency of data and the null hypothesis. Also note that changing the natural frequency numbers to standardized formats, such as percentages, destroys the nested categorizations, and thus the computational simplification, of natural sampling.) Thus, whereas it is pretty easy to create numerical frequencies which are not in a natural sampling framework, it is difficult to construct a natural sampling framework without reference to frequencies.

The consequences of confusions about how natural sampling and numerical frequencies are related to each other has led to a number of claimed novel discoveries, which are observed from the other side as “re-inventions.” One example of this is that the principles of natural sampling have been co-opted as something new and different. These situations require some clarification, which hopefully can be done in a relatively concise manner.

Subsequent to the description and application of a natural sampling structure in the original Gigerenzer and Hoffrage (1995) paper (which explicitly drew on the work by Kleiter (1994) in developing the natural sampling idea), the basic structure of natural sampling has been re-invented at least four times in the literature. Each time, the new incarnation is described at a level of abstraction which allows one to consider the structure independent of frequencies (or any other numerical format), but the natural sampling structure is unmistakable:

(a) Johnson-Laird et al. (1999) reintroduced the basic relevant principle of natural sampling as their “subset principle,” implying that ecological rationality researchers somehow missed this property: “The real burden of the findings of Gigerenzer and Hoffrage, (1995, p. 81) is that the mere use of frequencies does not constitute what they call a ‘natural sample.’ Whatever its provenance, as they hint, a natural sample is one in which the subset relations can be used to infer the posterior probability, and so reasoners do not have to use Bayes’ theorem.” Note also the confusion in this passage between the narrow definition of Bayesian reasoning as using Bayes’ theorem and the more general, psychologically relevant definition of Bayesian reasoning we clarified earlier in this paper. Girotto and Gonzalez (2001) continue from this point in their use of the “subset principle,” which is simply an abstraction of the natural sampling structure;
(b) Evans et al. (2000) proposed a process that involves “cueing of a set inclusion mental model,” rather than a natural sampling structure;
(c) Macchi (1995) and Macchi and Mosconi (1998) created the label of “partitive formulation” to describe the natural sampling structure; and
(d) Sloman et al. (2003) use the term “nested-set relations” rather than natural sampling, following Tversky and Kahneman (1983).

As this last re-invention noted, Tversky and Kahneman (1983) did discover that using frequencies sometimes improved performance (e.g., in their work on the conjunction fallacy), but they did not actually elaborate this observation into a theory; they only speculated that frequencies somehow helped people represent class inclusion.

Dissociating the natural sampling framework, claiming that it is something else, and then looking at the effects of numerical frequencies by themselves (without natural sampling or with malformed natural sampling) has allowed for all sorts of methodological and conceptual shenanigans. It is not interesting, either methodologically or theoretically, that making Bayesian reasoning tasks harder (by adding steps, using wordings which confuse people, switching numerical formats within the same problem) can decrease performance (see, Brase, 2002, 2008, 2009a,b, 2014 for further elaboration). Indeed, it is generally difficult to make strong theoretical claims based on people failing to accomplish a task, as there are usually many different possible reasons for failure.

In addition to multiple attempts to co-opt the concept of natural sampling there has been a notable attempt to co-opt the numerical format of frequencies, claiming that the facilitative effect of using frequencies is not actually about the frequencies themselves. Girotto and Gonzalez (2001) asserted that people actually can be good at Bayesian reasoning when given only probabilistic information. The probabilities used in this research, however, are of a peculiar type stated in whole number terms. For example:

Mary is tested now [for a disease]. Out of the entire 10 chances, Mary has ___ chances of showing the symptom [of the disease]; among these chances, ___ chances will be associated with the disease. (p. 274)

How many times was Mary tested? Once or ten times? If tested once, there is one “chance” for a result; if tested 10 times (or even if 10 hypothetical times are envisioned), then this is an example of frequency information. It seems odd to say that subjects are truly reasoning about unique events and that they are not using frequencies, when the probabilities are stated as *de facto* frequencies (i.e., 3 out of 10). Although Girotto and Gonzalez (2001) claim that “chances” refer to the probability of a single-event, it can just as easily be argued that this format yields better reasoning because it manages – in the view of the research participants—to tap into a form of natural frequency representation. This alternative interpretation was immediately pointed out (Brase, 2002; Hoffrage et al., 2002), but advocates of the heuristics and biases approach were not swayed (Girotto and Gonzalez, 2002).

In order to adjudicate this issue, Brase (2008) gave participants Bayesian reasoning tasks based on those used by Girotto and Gonzalez (2001). Some of these problems used the natural sampling-like chances wording. Other versions of this problem used either percentages (not a natural sampling format) or used a (non-chances) frequency wording that was in a natural sampling format. After solving these problems, the participants were asked how they had thought about the information and reached their answer to the problem. First of all, contrary to the results of Girotto and Gonzalez (2001), it was found that frequencies in a natural sampling structure actually led to superior performance over “chances” in a natural sampling structure. (The effect size of this result is actually similar to the Girotto and Gonzalez (2001) results, which were statistically underpowered due to small sample sizes.) More notably, though, the *participants who interpreted the ambiguous “chances” as referring to frequencies performed better than those who interpreted the same information as probabilities.* This result cuts through any issues about the computational differences between natural sampling frameworks versus normalized information, because the presented information is exactly the same in these conditions and requires identical computations; only the participants’ understanding of that information is different.

### Using Pictures to Aid Bayesian Reasoning

Generally speaking, pictures help Bayesian reasoning. Like the research on frequencies and natural sampling, however, there is disagreement on how and why they help. The ecological rationality account (Cosmides and Tooby, 1996; Brase et al., 1998) considers pictorial representations as helping because they help to tap into the frequency-tracking cognitive mechanisms of a mind designed by the ecology experienced over evolutionary history. That is, people have been tracking, storing, and using information about the frequencies of objects, locations, and events for many generations. Visual representations of objects, events, and locations should therefore be closer to that type of information with which the mind is designed to work. An alternative heuristics and biases account is that pictures help to make the structure of Bayesian reasoning problems easier to understand. This account of pictures helping because it enables people to “see the problem more clearly” is often tied to the co-opted and abstracted idea of natural sampling; the pictures help make the subset structure, the set-inclusion model, or the nested-set relations more apparent (e.g., Sloman et al., 2003; Yamagishi, 2003). Indeed, there are parallels here in the comparison of these two perspectives: the ecological rationality account proposes a more narrowly specified (and evolutionary based) account, whereas the heuristics and biases account favors a less specific (non-evolutionary) account.

Subsequent research (Brase, 2009a, 2014) has taken advantage of the fact that ambiguous numerical formats can be interpreted as either frequencies or as probabilities. By using the “chances” wording for the actual text and therefore holding the numerical information as a constant, while varying the type of pictorial representation, this research has been able to compare different types of pictorial aids against a neutral task backdrop. Brase (2009a) found that, compared to control conditions of no picture at all, Venn circles (which should facilitate the perception of subset relationship) did not help nearly as much as pictures of icon arrays (which should facilitate frequency interpretations of the information). Furthermore, a picture with intermediate properties – a Venn circle with dots embedded within it – led to intermediate performance between solid Venn circles and icon arrays. Subsequent research by Sirota et al. (2014b) took an interesting intermediate theoretical position, claiming that the heuristics and biases account predicted *no facilitation* of Bayesian reasoning from using pictures (contra Sloman et al., 2003 and Yamagishi, 2003). Their null findings of several different types of pictures failing to improve Bayesian reasoning are used to challenge the ecological rationality account, which they agree does predict an improvement with the use of pictures. A nearly concurrent publication replicated and extended the specific effects of Brase (2009a), however, casting doubt on the significance of the Sirota et al. (2014b) null findings. Brase (2014) found that roulette wheel diagrams (like those used in Yamagishi, 2003) led to performance similar to that of Venn diagrams, and that both realistic and abstract icon shapes significantly improved performance. Interpretation of the ambiguous numerical information as frequencies also improved Bayesian reasoning performance in all these conditions (replicating the findings of Brase, 2008), separate from the effects of the different picture types.

## Individual Differences in Bayesian Reasoning

There have been various claims that certain individual differences may moderate the often-observed frequency effect in Bayesian reasoning. Peters et al. (2006) demonstrated that numerical literacy (or numeracy)—an applicable understanding of probability, risk, and basic mathematics—moderated many classic judgment and decision making results, showing proof of concept that not all judgment and decision making tasks may be viewed the same by every individual. Specifically, Peters et al. (2006) showed that low numerates may benefit the most from number formats designed to aid comprehension of the information. The explanation proposed for these results can be summarized as a “fluency hypothesis”: that more numerically fluent people (higher in numerical literacy) are influenced less by the use of different numerical formats because they are quite capable of mentally converting formats themselves. In doing so, these highly numerate people utilize the numerical format best suited for the present task. Less numerically fluent people, on the other hand, are prone to work only with the numerical information as presented to them. This leaves them more at the mercy of whatever helpful or harmful format is given to them. Although Peters et al. (2006) did not assess Bayesian reasoning specifically, Chapman and Liu (2009) later brought the issue of numerical literacy to the topic of frequency effects in Bayesian reasoning tasks.

The story takes an interesting turn at this point, because although Peters et al. (2006) showed low numerates benefited most from a number format change to frequencies, Chapman and Liu (2009) showed instead that high numerates differentially benefited from natural frequency formatted Bayesian reasoning problems. Specifically they found that this frequency effect was only observed in highly numerate individuals, resulting in a statistically significant numeracy x number format interaction. Chapman and Liu (2009) pointed out that some other research is consistent with these results. In particular, Bramwell et al. (2006) provided different groups of participants with Bayesian reasoning problems framed as a test for a birth defect. The participants were either obstetricians, pregnant women and their spouses, or midwives. The effect of presentation format was assessed with a between-subjects manipulation, with some participants receiving naturally sampled frequencies and others receiving a single event probability format. Although the frequency effect was observed in their study, a closer examination showed that this effect was limited to obstetricians, whereas the midwives, pregnant women, and their spouses all showed equally poor Bayesian reasoning performance regardless of number format.

To the extent that obstetricians have somewhat higher numerical literacy, which is a plausible assumption, the Bramwell et al. (2006) results would be consistent with those of Chapman and Liu (2009). Both of these results, however, are inconsistent with the findings and the fluency hypothesis of Peters et al. (2006). Chapman and Liu (2009) proposed something akin to a “threshold” hypothesis regarding the interaction effect they found. This threshold hypothesis proposes that a certain level of numerical literacy is required for difficult problems (such as Bayesian reasoning tasks) before helpful formats (e.g., naturally sampled frequencies) are able to provide an observable benefit.

To assess this threshold hypothesis and the fluency hypothesis proposed by Peters et al. (2006), Hill and Brase (2012) systematically tested a variety of problem types with varying levels of difficulty and in different number formats, while also assessing numerical literacy with the standard measure used in this research (i.e., the General Numeracy Scale; Lipkus et al., 2001). These findings generally showed an absence of any interaction across several different problem types. Of most importance to the current paper, the Bayesian reasoning problems originally used by Chapman and Liu (2009) also failed to replicate the numeracy × number format interaction, causing some specific concern over the “threshold hypothesis” of Bayesian reasoning, and to a lesser extent the “fluency hypothesis” of judgment and decision making tasks in general. The one constant across these studies was a consistent main effect for numeracy and a consistent main effect for number format, with higher numerates performing better on Bayesian reasoning tasks, and participants given the natural frequencies format also performing better than those given single event probability versions.

Support for the findings of Hill and Brase (2012) were shown by Garcia-Retamero and Hoffrage (2013) who studied the Bayesian reasoning ability of doctors and patients in medical decision tasks. After fully crossing conditions by number format (natural frequencies and single event probabilities) and display (number only or pictorial representation), participants’ numeracy scores were also assessed. Garcia-Retamero and Hoffrage (2013) found the traditional frequency effect, just as in Hill and Brase (2012), and also an improvement in Bayesian reasoning performance by including a pictorial representation. Numeracy did not interact with the frequency effect, again consistent with the Hill and Brase (2012) findings and with the ecological rationality explanation of the frequency effect. Johnson and Tubau (2013) also partially replicated the lack of a numeracy × number format interaction, and found consistent improvement in Bayesian reasoning as a result of using natural frequencies, with the only exception being in very difficult problems, operationally defined by longer word length of the problem text. Johnson and Tubau (2013) proposed that both Chapman and Liu (2009) and Hill and Brase (2012) may be partially correct. When given long (“difficult”) problems, the numeracy × number format interaction was present, with low numerates showing a floor effect, and high numerates showing the benefit of natural frequencies, a finding consistent with the “threshold hypothesis” of Chapman and Liu (2009). However, with less difficult problems the numeracy × number format interaction disappeared, a finding in line with Hill and Brase (2012).

The above set of results led Johnson and Tubau (2013) to suggest a potential problem with evolutionary accounts proposed by various researchers (e.g., Cosmides and Tooby, 1996; Brase et al., 1998), in that there was not a frequency facilitation effect for the *very* difficult problems. The present authors, however, do not see this as a problem for an evolutionary account. We reach this conclusion because differences in *problem context* (e.g., problem difficulty, word count) that are assessed in terms of the written problem properties are only tenuously connected to evolved cognitive abilities. Cognitive mechanisms evolved to solve specific problems in specific environments. The perspective of ecological rationality, which is generally consistent with evolutionary psychology, is also built upon a similar premise (i.e., the fit between the structure of the environment and the design of the mind; Gigerenzer et al., 1999; Gigerenzer and Gaissmaier, 2011). By analogy, this situation can be compared to someone proposing that humans have an evolved ability to develop complex language. This proposal is not endangered by the observation that people (even highly literate people) find a college physics textbook difficult to read. Reading is a cultural invention which taps into our evolved language ability, and thus our ability to handle a particularly difficult written text is only tenuously connected to the evolved cognitive ability for human language.

More recent work on individual difference moderators of the frequency effect in Bayesian reasoning has only made the aforementioned research more perplexing. For instance, McNair and Feeney (2015) demonstrated a “threshold” type effect despite slightly different problem format manipulations. Specifically, McNair and Feeney (2015) assessed the differences between the standard format (single event probabilities) and a causal format (still single event probabilities, but with additional text describing a possible cause for false positive test results); previous research by Krynski and Tenenbaum (2007) demonstrated evidence that causal structures in problems could lead to improved Bayesian reasoning performance. In separate studies, McNair and Feeney (2015) found evidence for numerical literacy serving as a moderator of problem structure’s benefits on Bayesian accuracy, with the effect of problem structure only present in highly numerate individuals. Similar to the discussion of the threshold hypothesis of Chapman and Liu (2009), this observation of an apparent moderating relationship between privileged representational formats, and individual difference measures (e.g., numeracy, cognitive reflection) might be seen as damaging to evolutionary and ecological accounts. However, the same explanation as offered for the Chapman and Liu (2009) results can hold for the McNair and Feeney (2015) results: that performance near floor effect levels can resemble an interaction. In fact, performance in the McNair and Feeney (2015) studies *was* somewhat low (range: 3 to 32% in lowest to highest performing conditions).

Other recent research (Lesage et al., 2013; Sirota et al., 2014a) has addressed a commonly held assumption critics make about the “ecological rationality account”: if naturally sampled frequencies are a privileged representational format for an evolved statistical reasoning module, then the module must be “closed,” and automatic. Thus, any general cognitive traits (e.g., cognitive reflection), or any method of decreasing general cognitive capacity (e.g., cognitive load), should not significantly interfere with Bayesian performance, or the frequency effect. In general terms, this idea is the assumption of modular encapsulation (Fodor, 1983), which is still promoted by Fodor but actually not accepted by any prominent evolutionary psychology views (e.g., compare Fodor, 2000 and Barrett, 2005).

Although both groups of authors readily acknowledge the research conducted, and the reviews published, concerning the massive modularity hypothesis, there does seem to be some misunderstanding. For example, Barrett and Kurzban (2006, see specifically pp. 636–637), which is cited by some of the work mentioned above, discuss at length the misunderstandings about automaticity of evolved modules, and the method of using cognitive load induced deficits as evidence against evolved modules. Without getting too detailed, their arguments can be summarized by the following analogy: personal computers have a variety of specialized programs (modules). Few would argue that a word processor works as efficiently at storing and computing numerical data, as compared to a spreadsheet program. Thus, these programs are separate, and specialized. However, if I download 1,000 music files to my computer, the overall performance of those separate programs will suffer, at least with respect to processing time. Also, if I drain the battery power in my laptop, the programs will fail to operate at all. This observation does not lead directly to the conclusion that the programs are not specialized. It simply points to the conclusion that the programs require some overlapping general resources. The same conclusion should be made with respect to cognitive modules. The examples in this analogy are extreme instances of general situations which can impair the functioning of functionally specific modules, but the point holds. The question becomes not one of modular abilities being impervious to general resource constraints, but rather one of understanding *how* particular situational contexts influence the functioning of specific cognitive abilities.

In a different study of individual differences, Kellen et al. (2013) found the standard benefits of pictorial representations (Venn diagrams, in this case) in answering complex statistical tasks such as Bayesian reasoning. Furthermore, this general pattern interacted with measured spatial ability, which was independently assessed. In low-complexity problems, low spatial ability participants actually were hurt by pictorial representations, whereas high spatial ability participants demonstrated no difference between pictorial and text displays. However, in high-complexity problems, high spatial ability participants were aided in their understanding by the presence of pictorial representations, whereas low spatial ability participants saw no benefit. This last result is somewhat consistent with a threshold hypothesis, but there are many issues within these studies in need of deeper assessment. Further research is needed to clarify how different spatial ability levels are related to the use of different types of visual displays and if there is any relationship between spatial ability, numeracy, and the effects of naturally sampled frequencies.

Finally, there are differences in performance that are related to the incentive structures under which people are asked to do Bayesian reasoning tasks. Research participants who do Bayesian reasoning tasks as part of a college course (either through a research “subject pool” or as in-class volunteers) tend to perform less well than participants who are paid money for their participation (Brase et al., 2006). This same research also documented that participants from more selective universities generally performed better than those from less selective universities, most likely due to a combination of different overall ability levels and different intrinsic motivation levels to do academic-type tasks. Brase (2009b) extended this research to show that people whose payments were tied to performance (i.e., correct responses received more money) did even better than people who were given a flat payment for their participation. This is an important factor in, for example, understanding the very high level of Bayesian reasoning performance found by Cosmides and Tooby (1996; paid participants from Stanford University) versus the lower performance on the same task in Sloman et al. (2003; in-class participants from Brown University). In all cases, however, it should be noted that the relative levels of performance when varying the use of natural sampling, frequencies, and pictorial aids were consistent across studies. Absolute performance levels vary, but these methods for improving Bayesian reasoning remain effective.

## Conclusion

Overall, the literature on Bayesian reasoning is clear and straightforward in terms of *what* works for improving performance: natural sampling, frequencies, icon-based pictures, and more general development of the prerequisite skills for these tasks (i.e., numerical literacy, visual ability, and motivation to reach the correct answer). The more contentious topic is that of *why* these factors work to improve Bayesian reasoning. The balance of evidence favors the ecological and evolutionary rationality explanations for why these factors are key to improving Bayesian reasoning. This verdict is supported by multiple considerations which flow from the preceding review. First, the ecological rationality account is consistent with a broad array of scientific knowledge from animal foraging, evolutionary biology, developmental psychology, and other areas of psychological inquiry. Second, the ecological rationality approach is the view which has consistently tended to discover and refine the existence of these factors based on *a priori* theoretical considerations, whereas alternative accounts have tended to emerge as *post hoc* explanations. (To be specific, the facilitation effect of natural frequencies documented by Gigerenzer and Hoffrage (1995), the facilitative effect of pictorial representation documented by Cosmides and Tooby (1996), the effect of using whole objects versus aspects of objects documented by Brase et al. (1998), and the differential effects of specific types of pictorial aids in Bayesian reasoning documented by Brase (2009a, 2014) all were established based on ecological rationality considerations which were then followed by alternative accounts.) Third, the actual nature of the evidence itself supports the ecological rationality approach more than other accounts. For instance, in head-to-head evaluations of rival hypotheses, using uncontestable methodologies, the results have supported the ecological rationality explanations (e.g., Brase, 2009a). Furthermore, a quite recent meta-analysis (McDowell and Jacobs, 2014) has conclusively established the validity of the effect of naturally sampled frequencies in facilitating Bayesian reasoning, as described from an ecological rationality perspective.

Distressingly, some proponents of a heuristics and biases view of Bayesian reasoning have not engaged with the bulk of the above literature which critically evaluates this view relative to the ecological rationality view. As just one illustration, Ayal and Beyth-Marom (2014) cite the seminal work by Gigerenzer and Hoffrage (1995), yet ignore nearly all of the other research done from an ecological rationality approach in the subsequent nearly 20 years. Robert Frost (1919/1999) noted that people often say “good fences make good neighbors,” but that this is not necessarily a true statement:

Before I built a wall I’d ask to know

What I was walling in or walling out,

And to whom I was like to give offence.

Something there is that doesn’t love a wall,

In science, perhaps even more than in other domains of life, fences are *not* good. Willingness to engage openly, honestly, and consistently with the ideas one does not agree with should be a hallmark of scientific inquiry. Failing to do so is scientifically irresponsible.

In conclusion, the vast majority of studies in human Bayesian reasoning align well with evolutionary and ecological rationality account of how the mind may be designed. These accounts are theoretically parsimonious and established in a rich set of literature from a wide range of interrelated disciplines. Alternative explanations, however, tend to appeal to stripped down parts of this account, often losing clear predictive power in the process, which neglect the ecological and evolutionary circumstances of the human mind they purport to explain. That does not mean that the heuristic and biases account no longer has any validity. The intellectually invigorating component of this debate is that we do not fully understand all that is to learn about how people engage in (or fail to engage in) Bayesian reasoning. There is still much to learn about the possible environmental constraints on Bayesian reasoning (e.g., problem difficulty, number of cues), and how those constraints may be interwoven with individual differences (e.g., numerical literacy, spatial ability), and even different measures of specific individual differences (e.g., subjective vs. objective numeracy). We look forward to disassembling walls and integrating various perspectives, with the hope of more fully understanding how to improve Bayesian reasoning, and how those methods of improvement illuminate the nature of human cognition.

## Conflict of Interest Statement

The authors declare that the research was conducted in the absence of any commercial or financial relationships that could be construed as a potential conflict of interest.
